# Development of microsatellite loci and optimization of a multiplex assay for *Latibulus argiolus* (Hymenoptera: Ichneumonidae), the specialized parasitoid of paper wasps

**DOI:** 10.1038/s41598-020-72923-6

**Published:** 2020-09-30

**Authors:** Agata Kostro-Ambroziak, Anna Siekiera, Magdalena Czajkowska, Jan J. Pomorski, Hanna Panagiotopoulou

**Affiliations:** 1 Laboratory of Insect Evolutionary Biology and Ecology, Faculty of Biology, University of Bialystok, K. Ciołkowskiego 1J, 15-245 Białystok, Poland; 2 Museum and Institute of Zoology Polish Academy of Sciences, Wilcza 64, 00-679 Warsaw, Poland; 3 Division of Molecular Biology, Faculty of Biology, University of Bialystok, K. Ciołkowskiego 1J, 15-245 Białystok, Poland

**Keywords:** Biological techniques, Ecology, Evolution, Molecular biology, Zoology

## Abstract

Microsatellite loci are commonly used markers in population genetic studies. In this study, we present 40 novel and polymorphic microsatellite loci elaborated for the ichneumonid parasitoid *Latibulus argiolus* (Rossi, 1790). Reaction condition optimisation procedures allowed 14 of these loci to be co-amplified in two PCRs and loaded in two multiplex panels onto a genetic analyser. The assay was tested on 197 individuals of *L*. *argiolus* originating from ten natural populations obtained from the host nests of paper wasps. The validated loci were polymorphic with high allele numbers ranging from eight to 27 (average 17.6 alleles per locus). Both observed and expected heterozygosity values were high, ranging between 0.75 and 0.92 for H_O_ (mean 0.83) and from 0.70 to 0.90 for H_E_ (mean 0.85). The optimized assay showed low genotyping error rate and negligible null allele frequency. The designed multiplex panels could be successfully applied in relatedness analyses and genetic variability studies of *L*. *argiolus* populations, which would be particularly interesting considering the coevolutionary context of this species with its social host.

## Introduction

The ichneumonid wasp *Latibulus argiolus* (Rossi, 1790) of the subfamily Cryptinae (Hymenoptera: Ichneumonidae) is a specialized ectoparasitoid of social paper wasps (*Polistes* Latreille, 1802) with respect to its larvae and pupae^1,2^. Because parasitoids use only one host for their full larval development^3^, they are under strong natural selection that in turn leads to optimal exploitation of the host^4^. Therefore, the most important behaviour increasing the fitness of free-living female parasitoids is their oviposition decision, including the choice of the host and number of eggs laid on a specific host among other factors. A nest of paper wasps containing many potential hosts (various developmental stages of the wasp), can be attractive for the *L*. *argiolus* female allowing it to theoretically maximize its fitness. On the other hand, the strong defensive capabilities of *Polistes* colonies against intruders can influence the oviposition mode of *L*. *argiolus,* for example, by reducing the number of eggs laid in a single nest and in turn, by enlarging the number of host nests parasitized by this parasitoid. Furthermore, up to 34% to 78% of the host nests are destroyed by predators (such as birds and ants)^5^, that can lead to high *L*. *argiolus* mortality and lower its genetic variability. In turn, this process might cause a decrease in the fitness of this parasitoid and might function as an additional factor for females not to limit their reproduction to a single host nest. By linking the oviposition decision and genetic variability of *L*. *argiolus*, we can interpret the biology of this parasitoid in a broad ecological and evolutionary context. Investigations of the *L*. *argiolus* and paper wasp relationships may serve as a model for the coevolution of solitary parasitoid–social host interactions.

The marker of choice for many areas of population research, such as mating systems, kinship structure, demography or conservation genetics, are microsatellite loci^6–8^. The usefulness of these markers results from their codominance, high polymorphism, and abundance throughout the genome^9^. The high popularity of microsatellite loci, resulting in their frequent application in population genetics studies, is caused also by the beneficial proportion between the amount of information gained and the sum of financial costs and expended work efforts. Thus far, no such loci have been described for the *L*. *argiolus* species; therefore, the purpose of this study was to investigate these loci. Several methods for novel microsatellite marker development exist. One of them introduces third generation sequencing on a PacBio RSII platform. The described de novo loci must be verified first for reliable amplification and high polymorphisms. Microsatellites fulfilling the criteria of error free amplification, high genotyping quality, and polymorphisms may be applied for population genetic analysis. In studies applying microsatellites, at least over a dozen loci are required to obtain high statistical power of the calculations results^10,11^. Therefore, concurrent amplification of markers in multiplex polymerase chain reactions (PCR) is desirable because this procedure will significantly shorten the time of laboratory work and reduce the cost of analysis. Such assays need to be first optimized and validated to justify the use of the loci in future research projects^12–14^.

This study aimed to develop a set of novel microsatellite loci and then to optimize and verify the newly designed multiplexes that could be applied in population and evolutionary studies of the specialized ichneumonid parasitoid *L. argiolus* species.

## Material and methods

### Sampling and DNA extraction

The study material consisted of 213 *L. argiolus* specimens (126 diploid females and 87 haploid males). Specimens were obtained from 43 *Polistes* nests from ten wild populations in Poland: (1) Białystok-Lotnisko (53°05′59.5″N 23°09′37.6″E), (2) Haćki (52°50′05.5″N 23°10′57.9″E), (3) Kalitnik (53°00′54.5″N 23°42′54.7″E), (4) Krzyżanowice (50°27′12.4″N 20°33′29.1″E), (5) Mietlica (52°32′28.0″N 018°22′57.3″E, (6) Przewóz (52°28′51″N 18°23′25″E), (7) Skupowo (52°49′51.0″N 23°42′55.9″E), (8) Suraż (52°55′19.3″N 22°57′49.9″E), (9) Stawiany (50°35′30.6″N 20°37′19.7″E), and (10) Zalesiany (53°04′10.4″N 23°02′32.9″E). The wasp nests collected in the field (in May/June) were transferred into plastic cages (24 × 17 × 16 cm) and kept in the laboratory at temperatures of 23 to 27 °C under natural light:dark (L:D) conditions. The individuals of *L*. *argiolus*, emerging from the host nest in June/July, were frozen and preserved at − 20 °C. DNA was obtained from the hind legs using the DNeasy Blood & Tissue Kits (cat. no. 69506, Qiagen).

### Pacific biosciences RS platform sequencing

The PacBio library was constructed using 9 µg of genomic DNA consisting of 16 (12.5 µl) equally mixed female DNA samples. DNA was fragmented using miniTube (cat. no. 520064) in the Covaris System to a size of 1.5 to 3 kb according to protocol with minor modifications in which the intensity was set to 0.2 while the sonication time was reduced to 40 s. The shared sample was purified applying 0.6 × volume ratio of AMPure PB Beads (cat. no. 100-265-900, Pacific Biosciences). Furthermore, the 2 kb library was prepared using 750 ng of DNA according to the Pacific Biosciences protocol available online^15^ and using the SMRTbell Template Prep Kit 1.0 (cat. no. 100-259-100, Pacific Biosciences). The procedure included DNA damage and end repair followed by blunt ligation of hairpin adaptors at both ends of the DNA fragments. Failed ligation products were removed with *Exo*III and *Exo*VII enzymes. Purifications steps separating enzymatic reactions were performed by applying 0.6 × volume ratio of AMPure PB Beads. Size distribution of DNA fragments during the library preparation procedure and for the final library was checked by electrophoresis on 0.5% agarose gels. The resulting library was doubly purified and prepared for sequencing using DNA/Polymerase Binding Kit P6 v2 (cat. no. 100-372-700, Pacific Biosciences) and applying MagBeads Kit v2 (cat. no. 100-676-500, Pacific Biosciences) for loading onto the sequencer according to the protocol generated with Binding Calculator v.2.3.1.1 (available online: https://github.com/PacificBiosciences/BindingCalculator). Single molecule real-time testing (SMRT) was carried out on a PacBio RS II sequencer running two SMRT Cells 8Pac v3 (cat. no. 100-171-800, Pacific Biosciences) and a 360-min data collection mode.

Data gathered from sequencing were subject to the RS_ReadsofInsert.1 protocol analysis with default settings to generate reads of insert (ROI). The ROI reads were subsequently subjected to microsatellite analysis and primer design using msatcommander v.1.08^16^ with a threshold of at least eight or ten repetitions for tri- and tetra-nucleotide repeats, excluding mononucleotide repeats. Primer design parameters including the msatcommander option “combine loci” consisted of several parameters: (1) size of primers ranging from 18 to 22 bp, (2) annealing temperature (T_m_) ranging from 58 to 62 °C, (3) GC content ranging from 30 to 70%, and (4) amplicon product size range of 80 to 400 bp. Finally, only microsatellite sequences containing tri- or tetra-nucleotide motif with at least 10 repeats were used for marker selection and amplification test performance.

### Microsatellite marker selection and testing

Loci with the highest number of tandem repeats were chosen from the identified microsatellite sequences set. The DNA sequences of the newly developed loci were aligned against the NCBI database of already described nucleotide sequences using BLAST algorithms (BLASTN 2.10.1 + and BLASTX 2.10.1 + programmes)^17–19^. In the next step, loci were filtered for their optimal amplification performance by applying following criteria calculated in silico: (1) maximal PCR efficiency and no dimer formation, (2) difference in annealing temperature between primer pair below 2 °C, (3) minimal penalty score, and (4) sequence length of microsatellite ranging from 100 to 350 bp. For multiplex design purposes, the selected markers were further ordered according to the predicted PCR product size into three categories long (> 300 bp), medium (150–300 bp), and short (< 150 bp). The finally selected markers were amplified on DNA of 16 diploid females. Polymorphisms and the allele range of the chosen markers were tested using the universal primer labelling method^20,21^. The forward primers were tailed at the 5′ end with a CAC-sequence tag (5′-CACGACGTTGTAAAACGAC). This tag sequence was identical to four fluorescent dye-labelled universal forward primers (labelled with the dyes, including 6-Fam, Hex, Tamra, and Rox). The PCR reactions were performed in a final volume of 15 µl containing 9–227 ng of DNA (1 µl), 1 × MasterMix of DreamTaq Hot Start Green PCR Master Mix (cat. no. K9022, Thermo Fisher Scientific), 0.02 M tagged forward locus specific primer, 0.2 M locus specific reverse primer, and 0.2 M fluorescent labelled universal forward primers. The amplification conditions consisted of several steps: (1) initial denaturation 95 °C for 5 min, (2) 35 cycles of 30 s at 94 °C, (3) 90 s at 60 °C, (4) 40 s at 72 °C, and (5) a final elongation step of 10 min at 72 °C. Negative controls were included for each PCR reaction. Positive controls were not used as the DNA concentration and quality of all samples was satisfactory enough to permit successful PCR reactions. All loci were amplified separately, and the reaction efficiencies were visualized on 2% agarose gels. For loci in which no amplification products were observed, the amplifications were repeated by lowering the annealing temperature to 57 °C. PCR products were separated using an automated sequencer ABI 3500XL Genetic Analyzer applying GeneScan 600 LIZ as an internal lane size standard (cat. no. 4366589, Applied Biosystems). PCR products labelled with different fluorescent dyes were mixed for joint analysis. The resulting fragment sizes were read using GeneMapper v.4.1 (Applied Biosystems) software. Out of the tested set, only loci fulfilling following criteria were selected for multiplex panel optimization: (1) clear peak morphology, (2) no artefacts co-amplification, and (3) high number of alleles.

### Multiplex panel optimization

**Figure 1:**
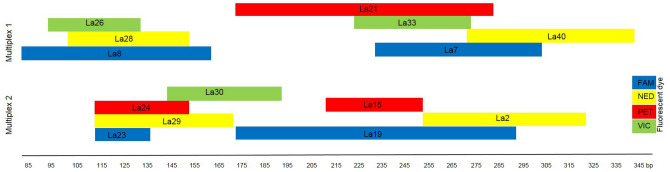
Two multiplex sets developed for simultaneous amplification of 14 microsatellite loci for *Latibulus argiolus*.

Out of 41 de novo described loci, 14 microsatellite markers were selected and organized into two multiplex PCR sets (seven loci each) by maximizing the number of loci labelled with the same dye (avoiding though allele overlap between loci) as shown in Fig. 1. The gaps between the neighbouring loci were set to the size of 66 to 137 bp according to the genotype data set of 16 females obtained during single microsatellite marker testing. The forward primer of each marker was fluorescence-labelled (Table 1, Fig. 1). Multiplex PCRs were performed with Labcycler Gradient (SensoQuest GmbH) in 5 µl reaction volume containing 2 µl genomic DNA (~ 20 ng), 1.7 µl Multiplex PCR 1 × MasterMix (cat. no. 206143, Qiagen), 0.3 µl mix of primers (cat. no. 450056, Life Technologies), and 1 µl RNase-free water (cat. no. 206143, Qiagen). Table 1 shows the final concentration of each primer pair. For each PCR reaction, a negative control was used as a sample to which no DNA was added. Each multiplex PCR started with an initial activation step of 95 °C for 15 min followed by 22 cycles of denaturation at 94 °C for 30 s, annealing at 57 °C for 90 s, extension at 72 °C for 60 s, and ended with a final extension at 60 °C for 30 min. The PCR products were mixed with 10 µl ultra-grade formamide (cat. no. 4311320, Applied Biosystems) and 0.2 µl GeneScan 500 LIZ size standard (cat. no. 4322682, Applied Biosystems), denatured at 95 °C for 5 min, rapidly cooled, and then subjected to fragment length analysis by using a four-capillary ABI 3130 Genetic Analyzer (Applied Biosystems). The fragment lengths of microsatellite alleles were estimated automatically using the AutoBin feature in GeneMapper 4.0 software (Applied Biosystems), checked, and then optionally corrected manually to avoid false alleles calling of the software. In addition, approximately 10% of the samples (N = 20) was randomly chosen, re-amplified, and genotyped in order to calculate the genotyping error of the optimized multiplex.

**Table 1:** Characterization of 14 microsatellite loci developed for *Latibulus argiolus* and organized in two multiplex sets (1, 2).

Locus/GenBank	Repeat motif	Primer sequences	Multiplex (Mix)	Fluorescent dye	µM	Size range	N_A_	H_E_	H_O_	PIC	*F*null	*F*null micro	P_HWE_
La7	ACG	(F) TTTGATTCGCTCGTTGGGTG	LaI (1)	6-FAM	0.3	237–307	17	0.84	0.84	0.81	− 0.003	− 0.003	0.278
MN531314		(R) CGACGTAGCACAACCCTTTC											
La8	ACG	(F) TCAATCGCGCGTACAAGAAC	LaI (1)	6-FAM	0.15	86–166	21	0.90	0.92	0.89	− 0.014	− 0.014	NE
MN531315		(R) GCGGTGACTGTTCTTCCAC											
La21	ACG	(F) GCGCTCGGTAAGTACGAAAC	LaI (1)	PET	0.375	176–280	27	0.84	0.79	0.83	0.034	0.033	0.084
MN531328		(R) ATCAGTTCGAAGCGTGTTGG				(176–234)	(25)						
La26	AAG	(F) GTTCCACGGCGAAAGAAGAG	LaI (1)	VIC	0.05	95–134	14	0.83	0.82	0.81	0.004	0.004	0.860
MN531333		(R) AGATATAACGGCGGGTCAGG					(13)						
La28	AGG	(F) GTACAATCGGAAGCGGAACC	LaI (1)	NED	0.1	101–150	14	0.87	0.82	0.86	0.030	0.029	NE
MN531335		(R) ACTCTTCTCGTTCGTCCGTC				(110–150)	(12)						
La33	AAG	(F) GTTCGGAAAGCATGGGACTG	LaI (1)	VIC	0.275	220–279	18	0.89	0.87	0.87	0.007	0.007	NE
MN531340		(R) GGATTCGTTTCGCCAGTTCC											
La40	ACG	(F) GAAGGACAACAAAGGCGGAC	LaI (1)	NED	0.5	277–344	22	0.88	0.82	0.86	0.031	0.030	0.951
MN531347		(R) AGCGAGAGAGGTTCATTCGG											
La2	ACG	(F) CGTTGTGGATCGTGTTCTACC	LaII (2)	NED	0.8	260–324	18	0.87	0.85	0.85	0.013	0.013	0.930
MN531309		(R) AATTCTTGCTACCGATCGCC											
La15	AGC	(F) TACTACGAACGAGGTGAGGC	LaII (2)	PET	0.8	213–251	13	0.86	0.91	0.84	− 0.033	− 0.034	0.042
MN531322		(R)AGGAAGGAACGAGAGAAGGC					(12)						
La19	AAG	(F) GCGCGCGGAAATTCAATATC	LaII (2)	6-FAM	0.15	177–299	27	0.89	0.79	0.88	0.059	0.056	0.061
MN531326		(R) ACTCCCGACAGCTGATTGAG					(23)						
La23	AAG	(F) CCCTTCGTCCGGATCTACAG	LaII (2)	6-FAM	0.1	114–136	8	0.70	0.76	0.65	− 0.059	− 0.064	0.124
MN531330		(R) ATATGGAGACTCTGCCGCTG											
La24	AAG	(F) ATCTTTATCTCGGGAGGCCG	LaII (2)	PET	0.1	113–158	16	0.78	0.75	0.75	0.029	0.028	0.001*
MN531331		(R) TTAATGGGCAGAAGACGGAG											
La29	AAG	(F) GCAGGGCGTCTTTCTCAATC	LaII (2)	NED	0.1	118–177	17	0.90	0.84	0.89	0.036	0.035	NE
MN531336		(R) TCTTTGGGTACGAGACGGAG											
La30	ACG	(F) CGGAAGTTCAAAGGTACCGC	LaII (2)	VIC	0.1	146–193	15	0.80	0.78	0.77	0.004	0.004	0.593
MN531337		(R) CTTCCGACAATGCGCTGTAC											
Overall							17.6 (16.9)	0.85	0.83	0.83			

*GeneBank* GeneBank accession numbers; *Fluorescent dye* dye used for primers labeling; *µM *optimized primer concentration; *Size range* observed size range in bp of the amplified alleles; *N*_*A*_ number of alleles; *H*_*E*_ expected heterozygosity; *H*_*O*_ observed heterozygosity; *PIC* mean polymorphic information content; *Fnull* null allele frequency (CERVUS 3.0.3); *Fnull micro* null allele frequency (MICRO-CHECKER 2.2.1; van Oosterhout values); *P*_*HW*_ Hardy–Weinberg probability test, *NE* not evaluated, *Statistical significance following sequential Bonferroni correction (*P* < 0.001). H_E_, H_O_, PIC, *F*null, P_HW_, as well as Size range and N_A_ values in brackets were calculated exclusively for diploid females (N = 110).

### Data analysis

The number of alleles (N_A_) and their size ranges for each of the preliminarily tested locus were calculated with GenAlEx v.6.503 programme^22^. The software MICRO-CHECKER 2.2.1^23^ was used for identifying possible genotyping errors (stuttering, large allele drop-out, false, and null allele frequencies) by performing 1,000 randomizations. We used CERVUS 3.0.3^24^ to estimate the number of alleles per locus (N_A_) and their size ranges. CERVUS 3.0.3 was also used to calculate the expected and observed heterozygosity (H_E_ and H_O_, respectively), the mean polymorphic information content (PIC), and the null allele frequency values (*F*null), and to estimate departures from Hardy–Weinberg equilibrium (HWE) with Bonferroni correction^24^ for evaluating the significance of HWE deviations. Linkage disequilibrium between loci was tested using GENEPOP v4.0^25^ with the Markov chain method (10,000 dememorization steps, 100 batches, 5,000 interactions) and Fisher’s exact test; a Bonferroni correction for multiple testing was then applied^26^. The described calculations were performed exclusively for diploid females (N = 110) with exceptions for the N_A_ and size range values, which were conducted for the whole sample set (N = 197), including haploid males (N = 87). Because the analysed females came from populations, in which the number of females was on average only 7.2, calculations were performed for the pooled sample of 110 diploid individuals.

## Results

### De novo sequencing of microsatellite loci

Library sequencing allowed us to obtain 1,133 Mb of raw data covering 65,556 reads for the jointly used SMRT Cells. The average sizes of the inserts were 2,241 and 2,464 (with quality of the inserts reads exceeding 0.92). The final output of reads length reached 151.4 Mb. Out of these sequences set, 48,515 ROIs were acquired and accumulated with 90,546,727 bp. The average ROI length equalled 1,874 bp, and the average number of passes was over 17 (17.74). The final sequence list consisted of 11,355 DNA sequences that were subjected to microsatellite loci searching. Sorting of microsatellites from this set allowed 415 sequences to be found that had an at least eight repetition threshold for which 401 primer pairs could be successfully designed. Altogether, 194 microsatellite loci covering tri- or tetra-nucleotide repeats at a number higher or equal to 10 were identified (Supplementary Table S1), for which 173 primers pairs could be designed (Supplementary Table S2). Only six tetra-nucleotide loci fulfilling the microsatellite selection criteria (out of these set) were detected (Supplementary Table S1). From this set, 41 tri-nucleotide loci, named LA1–LA41 were selected for PCR amplification and testing (Supplementary Table S3) and then deposited in GenBank under accession numbers MN531308–MN531348.

Out of 41 amplified markers tested in 16 diploid *L. argiolus* females, 40 (97.6%) were successfully amplified and were polymorphic (Supplementary Table S4). For four markers (LA1, 14, 16, and 38), the annealing temperature had to be lowered to 57 °C in order to obtain PCR products. Most of the markers (78%) amplified well and yielded clear and readable products. Twenty-four (60%) of the markers gave single peaks, while in the others 16 (40%), additional loci peaks impeding potential uncertainness in allele scoring were observed. These peaks consisted of stutter bands or after-peaks. In seven cases, two peaks differing by one bp in length were observed. The latter effect is caused in cases in which polymerase does not add adenine to the 3′ end of the newly synthesised strand during replication (resulting in so called − A and + A bands occurrence)^27^. BLASTX analysis showed no results for neither of sequence of the 41 tested loci. BLASTN results indicated that eight DNA sequences were similar to previously described DNA or cDNA sequences deposited in GenBank (Supplementary Table S5). However, in all results, the obtained query cover values were too low for convenient sequence annotation to the already described in GenBank genes or transcripts. This indicates that the selected loci are probably located in the non-coding regions of the *L. argiolus* genome. The exception could be locus La36 for which the query cover values exceeded 50% together with low E value scores and relatively high percentage identity of the aligned sequences for several insect species. In most of these cases BLAST search identified transcription factors sequences as the most probable results. The observed allele number in the tested loci ranged from 4 to 18 with 25 loci exhibiting at least 10 alleles. The best amplified, readable, and most polymorphic loci (Supplementary Table S4) were considered for multiplex development. LA11 was excluded from this set since it exhibited difficulties in raw read rounding, which could have been caused by its hypervariability (N_A_ = 18).

### Development and characterization of the microsatellite multiplex assay

For multiplex optimization purposes, 14 loci were selected and organized into two multiplex panels (Table 1, Fig. 1). The combined microsatellite primers pairs in these assays successfully amplified the chosen loci to yield peaks that were clear and easy to score. For most of the loci, the initial primer concentration had to be adjusted to achieve balanced amplification of the markers (Table 1). We did not detect any evidence of genotypic errors using the MICRO-CHECKER 2.2.1 software, a finding that was also proven by the low frequencies of null alleles explored by CERVUS 3.0.3 (Table 1). The amplification success of the multiplexes was high, reaching 197 (100%) of the microsatellite profiles. Robustness of the optimized assay was further verified by obtaining 100% correctly genotyped samples for 20 duplicated samples (~ 10% of the studied samples set as recommend by Pompanon et al.^27^). No significant linkage disequilibrium among any pair of the loci under study after Bonferroni correction (adjusted value of *P* < 0.004) was found, indicating that the studied loci were most probably not linked. Deviations from Hardy–Weinberg equilibrium were significant (*P* < 0.001) for only one (La24) of the 14 loci tested after Bonferroni correction (Table 1).

The average number of alleles (N_A_) per locus was 17.6, ranging from 8 in locus La23 to 27 in loci La19 and 21. The set of diploid female samples was characterized by the highly observed heterozygosity (mean H_O_ = 0.83) ranging from 0.75 in locus La24 to 0.92 in locus La8 in addition to the highly expected heterozygosity (mean H_E_ = 0.85) ranging from 0.70 in locus La23 to 0.90 in loci La8 and 29. The mean polymorphic information content (PIC) ranged from 0.65 in locus La23 to 0.89 in loci La8 and 29 (Table 1).

## Discussion

In this study, we successfully describe new loci and optimization of a multiplex assay for *L. argiolus*, the highly specialized parasitoid of social paper wasps. PacBio RSII platform sequencing allowed for reliable discovery of microsatellite markers. This method has been proven to perform very well in de novo microsatellite description and was recently often applied^28–30^. Application of this method is especially useful in non-model organisms in which no information about genomic data exists. In such cases, it also outperforms other methods by being more efficient and accurate^30,31^. This method is also relatively cost and time efficient compared to the classical methods of microsatellite development^9,28^. Out of 11,355 DNA sequences, approximately 1.7% contained tri- or tetra-nucleotide motifs exceeding ten repeats. Tri- and tetra-nucleotide loci were selected as they were proven to cause less difficulties in scoring and rounding^32^. This finding is important, especially since insects are considered problematic for microsatellite isolation and genotyping and are often biased with particularly high frequencies of null alleles^9,33,34^. Tri- and tetra-nucleotide loci have been suggested to be less frequent than di-nucleotide in the genome; however, they are less prone to amplifications errors, especially stuttering^29^. For this reason, primers and in silico amplifications were very carefully chosen. Sequences with high repeat numbers were selected as it was proven that such loci have higher polymorphism due to higher mutation rate caused by polymerase slippage^35^.

The amplification success of the designed markers was very high (97.6%), and most of the loci amplified well, which points to the high quality of the RSII sequencing and well-defined parameters for primers design^28–31^. The relatively long reads (on average, exceeding 2 kb) facilitated the design of primers that enabled high PCR performance^28^.

The approach of applying universal primer labelling^20^ has been reported to be very efficient and cost effective in other studies^21,32,36^. In our current study, it was also proven to be a very useful and suitable method for primer labelling. The selected markers amplified well, and in most cases, produced (78%) clear bands and in 60% produced single peaks that permitted confident reading. Almost all of the tested markers were polymorphic, which may have been the consequence of selecting sequences with more than ten repeats. The rate of polymorphic loci in conventional methods of de novo microsatellite isolation may be as low as 30%^30,35^. This finding indicates that appropriate microsatellite sequences were selected for amplification tests in single specimens. This finding may also point to a high intrapopulation polymorphism of the studied species. However, a trade-off between high variability and possible artefacts may exist. The extremely polymorphic markers may be biased with high null allele frequency as a consequence of high a mutation rate in these loci^9,27,33^. This bias could have occurred in the LA11 locus. For multiplex optimization, direct fluorescent labelling was applied to reduce primer-dimer formation and enhance the PCR efficiency^32^, which was 100% in the studied sample set.

Our data show that the 14 microsatellite loci selected for assay development are reliable markers for genetic analyses of *L*. *argiolus* individuals with a good quality of detection, high polymorphism, and low frequency of null alleles. It is true that one of the loci was not in HWE, but it should be emphasized that the HWE test was performed for pooled diploid individuals that came from different populations resulting most probably in Wahlund effect. We believe that the presented tool will be very useful in *L*. *argiolus* studies, especially in the context of its life history strategies after considering co-evolution of this haplodiploid parasitoid with its social host. Because oviposition decisions of female parasitoids primarily influence their fitness, we hypothesize that female of *L*. *argiolus* lays more eggs in larger nests of the paper wasp and exhibits adaptive sex ratio manipulation in response to the number of her offspring produced in a single nest of the host. The previously mentioned hypotheses could be now addressed by applying the developed marker set that would allow determination of kinship and could also be a very good tool for the rapid identification of sex of preimaginal stages of *L*. *argiolus*. We believe that application of microsatellite markers will yield many answers concerning the biology and genetic structure of this specialized parasitoid. Additionally, the deposited sequence data may be subject to further microsatellite searches and testing in other closely related species of this interesting group of parasitoids.

## Supplementary information

Supplementary Information.

## Data Availability

All data generated during this study are included in this published article and its supplementary information files.
